# Socioeconomic Status and Prognosis of Patients With ST-Elevation Myocardial Infarction Managed by the Emergency-Intervention “Codi IAM” Network

**DOI:** 10.3389/fcvm.2022.847982

**Published:** 2022-04-25

**Authors:** Helena Tizón-Marcos, Beatriz Vaquerizo, Josepa Mauri Ferré, Núria Farré, Rosa-Maria Lidón, Joan Garcia-Picart, Ander Regueiro, Albert Ariza, Xavier Carrillo, Xavier Duran, Paul Poirier, Mercè Cladellas, Anna Camps-Vilaró, Núria Ribas, Hector Cubero-Gallego, Jaume Marrugat

**Affiliations:** ^1^Hospital del Mar, Servicio de Cardiología, Barcelona, Spain; ^2^Grupo de Investigación Biomédica en Enfermedades del Corazón, Barcelona, Spain; ^3^IMIM (Instituto Hospital del Mar de Investigaciones Médicas), Barcelona, Spain; ^4^Centro de Investigación Biomédica en Red Enfermedades Cardiovasculares, Instituto de Salud Carlos III (ISCIII), Madrid, Spain; ^5^Facultat de Medicina, Universitat Autònoma de Barcelona, Bellaterra, Spain; ^6^Hospital Universitari GermansTrias I. Pujol, Servicio de Cardiología, Badalona, Spain; ^7^Departament de Salut, Generalitat de Catalunya, Barcelona, Spain; ^8^Hospital Universitari de la Valld'Hebron, Servicio de Cardiología, Barcelona, Spain; ^9^Hospital de la Santa Creu I. Sant Pau, Servicio de Cardiología, Barcelona, Spain; ^10^Hospital Clínic i Provincial, Servicio de Cardiología, Barcelona, Spain; ^11^Hospital Universitario de Bellvitge, Servicio de Cardiología, L'Hospitalet de Llobregat, Barcelona, Spain; ^12^AMIB, Assessoria Metodològica i Bioestadística, Barcelona, Spain; ^13^Insititut Universitaire de Cardiologie et Pneumologie de Québec, Québec, QC, Canada

**Keywords:** ST-elevation myocardial infarction, reperfusion, primary percutaneous coronary intervention, mortality, inequalities

## Abstract

**Background:**

Despite the spread of ST-elevation myocardial infarction (STEMI) emergency intervention networks, inequalities in healthcare access still have a negative impact on cardiovascular prognosis. The Family Income Ratio of Barcelona (FIRB) is a socioeconomic status (SES) indicator that is annually calculated. Our aim was to evaluate whether SES had an effect on mortality and complications in patients managed by the “Codi IAM” network in Barcelona.

**Methods:**

This is a cohort study with 3,322 consecutive patients with STEMI treated in Barcelona from 2010 to 2016. Collected data include treatment delays, clinical and risk factor characteristics, and SES. The patients were assigned to three SES groups according to FIRB score. A logistic regression analysis was conducted to estimate the adjusted effect of SES on 30-day mortality, 30-day composite cardiovascular end point, and 1-year mortality.

**Results:**

The mean age of the patients was 65 ± 13% years, 25% were women, and 21% had diabetes mellitus. Patients with low SES were younger, more often hypertensive, diabetic, dyslipidemic (*p* < 0.003), had longer reperfusion delays (*p* < 0.03) compared to participants with higher SES. Low SES was not independently associated with 30-day mortality (OR: 0.95;9 5% CI: 0.7–1.3), 30-day cardiovascular composite end point (OR: 1.03; 95% CI: 0.84–1.26), or 1-year all-cause mortality (HR: 1.09; 95% CI: 0.76–1.56).

**Conclusion:**

Although the low-SES patients with STEMI in Barcelona city were younger, had worse clinical profiles, and had longer revascularization delays, their 30-day and 1-year outcomes were comparable to those of the higher-SES patients.

## Introduction

Ischaemic heart disease is the leading component of cardiovascular disease (CVD), which is the first cause of mortality and morbidity worldwide (1, 2). Loss in quality-of-life and mortality due to cardiovascular disease has globally declined because of the implementation of evidence-based treatments and early and efficient management of acute coronary syndromes (ACSs), as well as appropriate drugs for secondary prevention and cardiac rehabilitation (3). Improvements in morbidity and survival after an ACS have spread unevenly throughout world regions, and they are more developed and implemented often in high-income countries (4). These differences may be partially explained by varying ACS emergency management modalities and population epidemiologic settings, including the influence of socioeconomic status (SES).

Multiple studies have shown that SES, measured by education level, occupation, or income, is associated with cardiovascular risk factors and outcomes after acute myocardial infarction (5–7). Even in high-income countries, poor socioeconomic circumstances may limit access to and increase delays in revascularization procedures, resulting in larger infarct size, lower compliance to secondary prevention drugs, and in general, poor application of and compliance with specialized state-of-the-art cardiac treatments (8, 9).

Implementation of coordinated and standardized treatment networks for acute ST-elevation myocardial infarction (STEMI) increases detection, decreases treatment times, and increases survival in all kinds of socioeconomic environments (6, 10).

The aim of this study was to analyze whether low SES determines more 30-day complications and 1-year all-cause mortality in patients with STEMI managed by the “Codi Infart Agut de Miocardi amb Elevació ST (Codi IAM)” emergency STEMI care network in Barcelona in a contemporary cohort from a European southern city.

## Materials and Methods

### Population

In this retrospective cohort study, we included patients from the registry of the “Codi IAM” emergency management network diagnosed with STEMI from 2010 to 2016 who were residents of Barcelona. The registry includes, in addition, cardiac arrest or patients who died with an initial electrocardiogram showing ST-segment elevation or patients with a new left bundle-branch block. Barcelona patients were treated in other facilities outside Barcelona and non-residents were excluded.

The “Codi IAM” STEMI emergency management network was launched in 2010 in a region of 7.6 million inhabitants. The three main objectives of the network were (i) to increase the rate of reperfusion therapy among patients with STEMI, (ii) to achieve primary percutaneous coronary intervention (PPCI) in less than 120 min from the first contact with the health system (3), and (iii) to monitor the results of the network with a registry. The city of Barcelona was the first to be completely covered by the network because of its high-density population. Three PPCI hospitals that are open 24 h/7 days a week and one hospital with daytime PPCI cover the city's STEMI management needs. Updated operating details of the network have been recently published by our group (11). Basically, an emergency medical service (EMS) is responsible for detecting patients with STEMI and coordinates facilities (EMS ambulances and helicopters, with PPCI hospitals).

ST-elevation myocardial infarction was defined according to current guidelines (3). Fibrinolysis was only considered if expected delays in PPCI treatment were not acceptable and there were no contraindications. Percutaneous coronary intervention (PCI) post-fibrinolysis was initially only considered in the absence of effective reperfusion. The local health system provides universal health coverage; in addition, it provides a partial subsidy for medication costs depending on patients' income and working status.

The registry of the “Codi IAM” activity started in 2010 and includes demographic, clinical, therapeutic, and discharge data collected in an electronic form from patients with STEMI occurring within the previous 12 h. Epicardial coronary flow in the culprit STEMI artery is graded according to Thrombolysis in Myocardial Infarction (TIMI) flow grade (12), and reperfusion is considered optimal when TIMI 3 flow is obtained in the culprit lesion. Data collection was extended to new variables in 2012 (acute pulmonary edema, number of diseased vessels, and TIMI flow) and in 2015 (hypertension, dyslipidemia, smoking status, previous stroke, previous treatment, and type and number of stents). Bleeding was included only in cases requiring transfusion.

The Family Income Ratio of Barcelona (FIRB) is an indicator of the mean income ratio of inhabitants of the 73 districts in Barcelona city and shows imbalances relative to the mean value of the city, which is set at 100. The FIRB has been calculated annually since 2007 by the Technical Office of the Barcelona City Council, and it can be accessed online (13). This indicator combines five concepts: i) ratio of university graduates, ii) ratio of unemployed to employable inhabitants, iii) the number of vehicles per inhabitant, iv) engine power of new vehicles acquired, and v) price of second-hand housing. FIRB was assigned to patients according to their address and district affiliation, and it has been used as a surrogate of socioeconomic status (SES) in this study. The patients were assigned to three SES classes according to the FIRB classification used by the Technical Office: low SES (low and very low SES corresponding to FIRB values below 80), mid SES (mid-low and mid-high corresponding to FIRB values from 81 to 125), and high SES (high and very high SES corresponding to FIRB values from 126 to 159) (13).

Mortality data of the patients were obtained from the National Mortality Registry. The quality of the data included in the registry is periodically verified by external audits.

### End Points

Primary assessment end points were 30-day mortality, a 30-day composite end point including death, ventricular fibrillation, pulmonary edema, or cardiogenic shock during admission, and 1-year all-cause mortality in 30-day survivors.

### Statistical Analysis

Categorical variables are shown as numbers and percentages; continuous variables are shown as mean and SD or as median and interquartile range, or as specified otherwise.

Patient characteristics and treatment times were compared according to each end point. Student's *t*-test or Mann-Whitney *U-*test was conducted for continuous variables and Chi-squared test, Fisher exact test, or ANOVA for categorical variables; *p*-tendency was used in all comparisons. A *p*-value < 0.05 was considered statistically significant. The association between SES and 30-day death or complications was evaluated with odds ratios (ORs) calculated using logistic regression models, and 1-year mortality in 30-day survivors was evaluated with hazard ratios (HRs) using Cox proportional-hazards regression models. The models were adjusted considering confounding variables associated with STEMI prognosis, which had <8% missing values: age, sex, diabetes mellitus, recruitment year, type of initial care, place of treatment, time from electrocardiogram to PPCI, and Killip class. Kaplan Meier survival curves were fitted for the three SES categories, and log-ranked *p*-values were used for comparison.

The Patients were assigned to the hospital where they spent most of their hospital stay.

In order to calculate 1-year mortality, the mortality of patients admitted in 2016 was analyzed up to 2017. Analyses were performed using SPSS software version 24.0. This project was approved by the ethics committee of Hospital del Mar (2020/9056), and all data are anonymous. The project was approved by the Scientific Committee of “Codi IAM”. Procedures and data collection complied with the Declaration of Helsinki and Spanish data protection laws.

## Results

**Figure 1:**
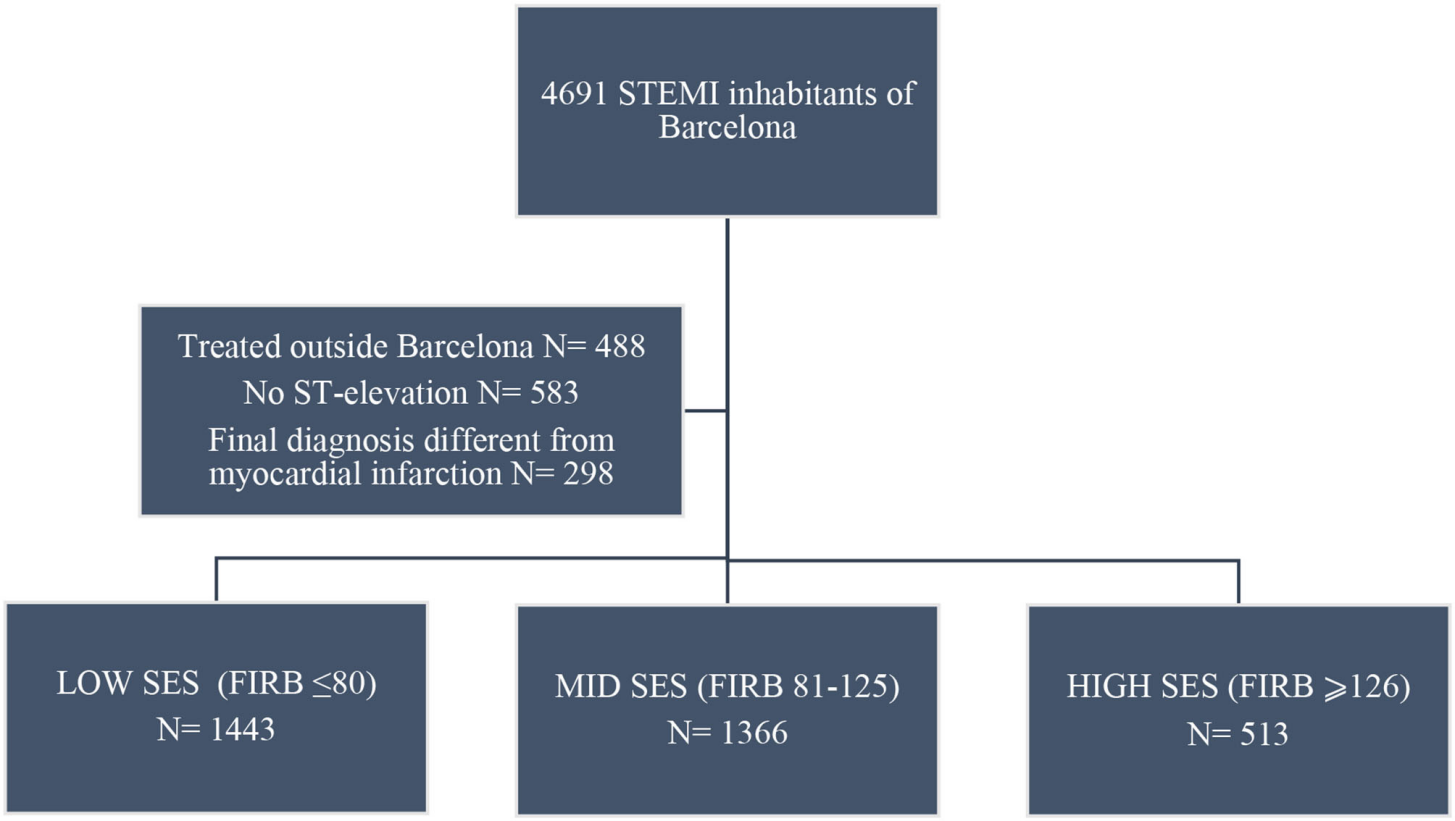
“Codi IAM” ST-elevation myocardial infarction (STEMI) patient inclusion flowchart.

The distribution of patients with STEMI treated in Barcelona city according to their SES is shown in Figure 1, in Figure 1: 43.4% had low SES, 41.1% had mid SES, and 15.4% had high SES. The annual hospitalization rate of patients with STEMI is shown in Supplementary Material 1. The number of patients with STEMI treated from the lowest SES was higher than the mean rate during the 7 years of the study.

Patients with low SES were younger, had a higher prevalence of cardiovascular risk factors, and were more often initially cared for by general practitioners (Table 1). In addition, they had a more deleterious cardiovascular risk factor profile than patients with higher SES. There was no difference in Killip class at STEMI presentation by SES.

**Table 1:** Baseline clinical characteristics according to socioeconomic status (SES) classification.

	**All *N =* 3,322**	**Low SES ≤80** ***N =* 1,443**	**Mid SES 81–125** ***N =* 1,366**	**High SES ≥126** ***N =* 513**	***P*-value**
Age, years	65.3 ± 13.5	64.4 ± 13.9	65.8 ± 13.3	66.6 ± 13.0	0.002
Women,%	26.3	27.0	25.9	25.3	0.720
Smokers,%^*^	26.5	27.1	27.0	23.2	0.460
Hypertension,%^*^	40.8	43.0	41.8	30.4	0.024
Dyslipidemia,%^*^	32.1	35.2	31.9	22.5	0.008
Diabetes mellitus,%	20.7	23.3	20.2	14.4	<0.005
Previous stroke, %^*^	2.6	2.9	2.2	2.9	0.790
Oral anticoagulant,%^*^	1.9	2.5	1.0	2.9	0.720
Antiplatelet drugs, %^*^	8.0	9.6	5.9	8.7	0.280
		**First contact**			
General practitioner, %	15.1	18.9	13.4	9.0	<0.012
Emergency medical system, %	47.7	49.1	44.8	51.1	
		**Complications**			
Mechanical ventilation, %	6.3	5.2	7.5	6.0	0.150
Ventricular fibrillation, %	7.4	6.4	8.4	7.2	0.240
Atrial fibrillation, %	1.4	1.9	1.1	0.8	0.026
AV blockade, %	4.9	4.4	5.6	4.5	0.550
Pulmonary edema, %^**^	2.2	1.5	3.1	1.4	0.430
Cardiogenic shock, %	6.4	5.9	6.8	6.4	0.480

*AV Blockade, atrioventricular blockade*.

* *Data since 2015, N = 991*.

** *Data available since 2012. N = 2416*.

Patients received PPCI as the first treatment in more than 93% of the cases but with no significant differences according to SES. Patients with higher SES had significant epicardial coronary disease significantly less often than those with mid and low SES (Table 2). Patients with lower SES had a tendency to be less often treated with drug-eluting stents (DESs) than those with mid and higher SES (55.1 vs. 57.5 vs. 69%, respectively, *p* < 0.35). Supplementary Material 2 shows differences in patients treated with DESs vs. bare-metal stents (BMS). DESs are significantly less used in the lower SES group; nevertheless, this difference is not greater than one unit.

**Table 2:** Primary reperfusion procedure in the “Codi IAM” network during the period 2010–2016 according to SES.

	**All *N =* 3,322**	**Low SES ≤80** ***N =* 1,443**	**Mid SES 81–125 *N =* 1,366**	**High SES ≥126** ***N =* 513**	***P*-value**
Coronary angiogram without PCI, %	5.0	4.6	5.5	5.0	0.640
PPCI, %	93.4	94.2	92.7	92.5	
Thrombolysis, %	0.2	0.3	0.2	0.2	0.240
Initial TIMI 0, %^*^	68.3	66.3	69.0	72.2	0.035
Initial TIMI 3, %^*^	11.0	12.1	10.2	9.6	0.120
Final TIMI 0, %^*^	2.1	2.2	1.6	3.0	0.630
Final TIMI 3, %^*^	91.6	92.1	91.5	90.6	0.390
No significant epicardial coronary disease, %^*^	5.3	4.3	5.5	7.6	0.023
3-vessel disease, %^*^	15.0	15.2	15.1	14.0	0.670
Left main disease, %^*^	4.2	3.9	4.2	5.2	0.300
Stents used^**^					
Only BMS use, %	40.6	43.5	39.8	28.6	
Only DES use, %	57.7	55.1	57.5	69	0.353
Both BMS and DES use, %	1.93	1.45	2.65	2.38	
Anterior STEMI, %	41.8	42.4	40.6	43.1	0.890
Inferior STEMI, %	43.8	42.2	45.2	44.6	0.180
Bleeding with transfusion, %	0.6	0.7	0.3	1.0	0.920

* *Data available since 2012*.

** *Data available since 2015. PPCI, Primary percutaneous coronary intervention; TIMI, thrombolysis in myocardial infarction flow; BMS, bare metal stent; DES, drug-eluting stent; STEMI, ST-elevation myocardial infarction*.

Patients with lower SES had longer delays regarding time from symptom onset to diagnosis (first electrocardiogram) than those with highest SES (Table 3), and longer times from symptom onset to PPCI hospital than the mid and high SES groups. Electrocardiogram-to-open-artery times showed a decreasing trend with SES. Longer ischemic times were observed in patients with lower SES. Accordingly, ratios of PPCI in less than 120 min were higher in those patients with higher SES compared to those with lower SES.

**Table 3:** Delays in initial medical care, diagnosis, and revascularization according to SES during the period 2010–2016.

	**All *N =* 3,322**	**Low SES ≤80** ***N =* 1,443**	**Mid SES 81–125** ***N =* 1,366**	**High SES ≥126** ***N =* 513**	***P*-value**
OS-ECG, min	82 [43–183]	85 [45–185]	85 [45–187]	75 [35–166]	0.003
ECG-Open Artery, min	85 [67–115]	87 [69–117]	83 [65–115]	81 [63–105]	<0.005
OS-Arrival to hospital, min	130 [82–240]	135 [85–240]	128 [80–254]	120 [75–225]	0.033
OS-Open Artery, min	184 [127–305]	190 [133–310]	180 [125–312]	165.5 [118–269]	0.003
PPCI <120 min from ECG, %	78.7	77.3	78.6	83.0	0.034
30-day mortality, %	7.7%	7.6%	8.3%	6.6%	0.430
30-day composite, %^**^	19.9%	19.4%	20.7%	19.1%	0.600
1-year mortality, %^*^	4.7%	5.2%	4.9%	5.1%	0.810

*OS, onset of symptoms; ECG, electrocardiogram; Min, minutes, median [interquartile range]*.

* *in 30-day survivors*;

** *death, pulmonary edema, cardiogenic shock, or ventricular fibrillation*.

**Figure 2:**
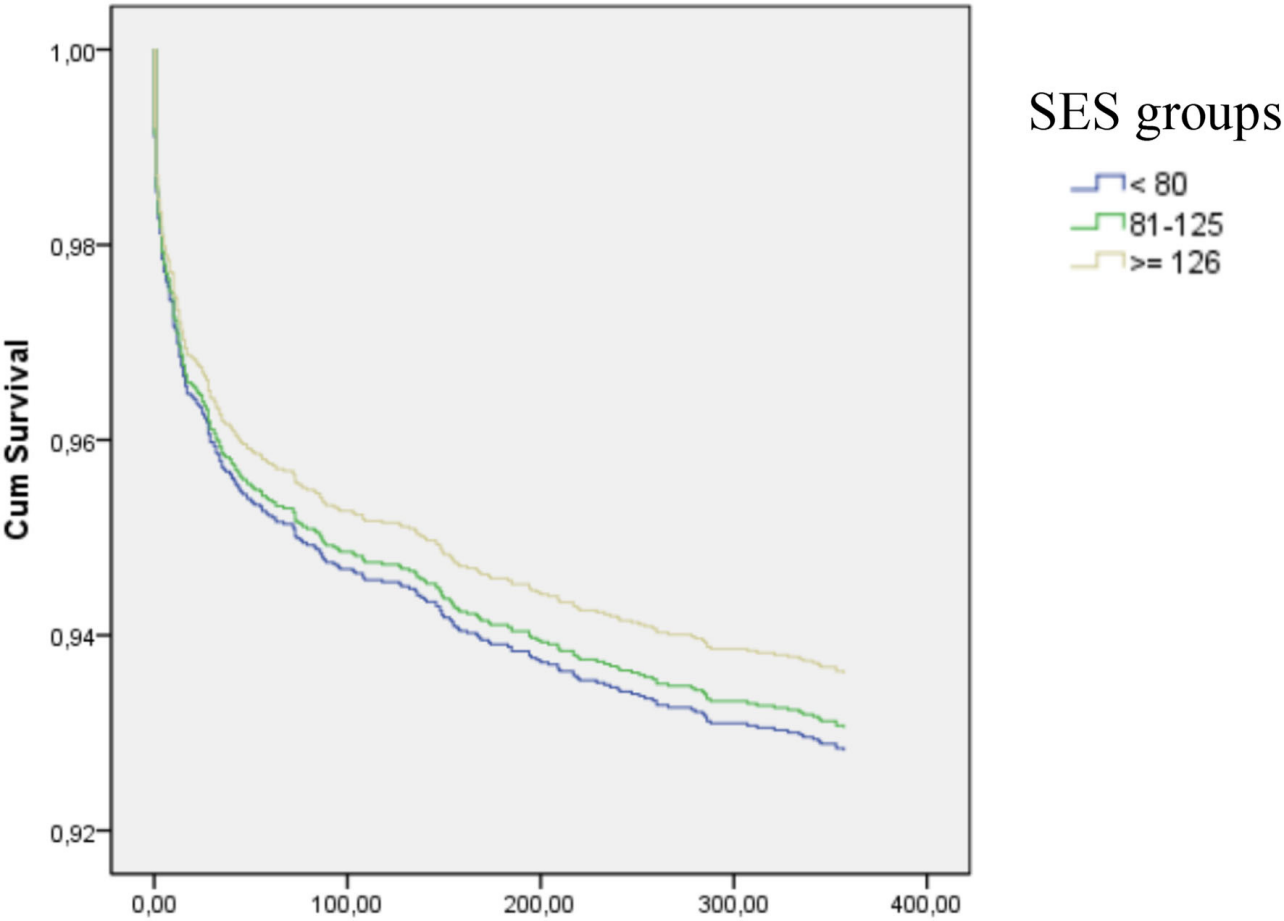
Cumulative survival according to socioeconomic status (SES). Global log-ranked *p*-values = 0.79; mid SES vs. low SES = 0.78; high SES vs. low SES = 0.49.

In Table 4, we show that 30-day mortality, 30-day cardiovascular complications, and 1-year mortality did not differ between the SES groups after adjusting for confounding factors. There were no significant differences in survival curves at 1-year according to SES (Figure 2).

**Table 4:** Low-SES patients with ST-elevation myocardial infarction (STEMI) adjusted odds ratio (OR) of 30-day mortality (model 1); a 30-day composite end point (death, ventricular fibrillation, acute pulmonary edema, or cardiogenic shock) (model 2); and hazard ratio (HR) of 1-year mortality in 30-day survivors (model 3) in the “Codi IAM” network during the 2010-2016 period.

**MODEL 1: 30-day mortality risk for STEMI patients in the whole cohort**		
***N** **=*** **3,078**	OR	95% CI
*Low SES*	0.95	0.70–1.30
**MODEL 2: 30-day composite endpoint risk for STEMI patients in the whole cohort**		
***N** **=*** **3,078**	OR	95% CI
*Low SES*	1.03	0.84–1.26
**MODEL 3: One-year mortality risk for STEMI patients in the whole cohort in 30-day survivors**		
***N** **=*** **2,720**	HR	95% CI
*Low SES*	1.09	0.76–1.56

*Models 1 and 3 were adjusted for age, sex, diabetes mellitus, Killip III/IV vs. I/II, first medical care, coronary percutaneous intervention, hospital, year of treatment, time from electrocardiogram to coronary percutaneous intervention (>120 min), and type of initial medical care. Model 2 was adjusted for age, sex, diabetes mellitus, first medical care, coronary percutaneous intervention, hospital, year of treatment, time from electrocardiogram to coronary percutaneous intervention (>120 min), and type of initial medical care*.

## Discussion

Low-SES patients with STEMI treated in Barcelona by the “Codi IAM” emergency network have a higher burden of cardiovascular risk factors and longer treatment delays than patients with higher SES. Despite this, they have similar mortality or cardiovascular complication rate at 30 days and similar 1-year mortality.

Recent STEMI guidelines have emphasized the importance of performing research addressing gaps in knowledge and the need for observational data and real-world evidence to ensure high-quality rapid and equal care for patients with STEMI in geographic areas (14). Previous studies from the late 1990s and early new century showed that SES was associated with both short- and long-term mortalities after ACS (15) and that an inverse trend in survival after myocardial infarction was also observed with SES levels in district-based studies (16, 17). However, these data come from heterogeneous studies, different time frames and may reflect inequalities in disease incidence, access to healthcare systems, and poor compliance with secondary prevention medications (18). Moreover, different SES indicators were used in previous studies: household income (14), highest educational level (15), geographical area (18), racial aspects (16), and various composite indexes (17, 19–21). Composite indexes can include varying combinations of household income, educational level, unemployment rate, vehicle ownership and engine power, prices of housing, the proportion of families with > 4 children, community services, environmental conditions, and crime level. These composites indexes reflect a multilevel stratum of social, educational, and economic data, which can give a better picture of concurring effects that determine health, acute and chronic treatments, and follow-up compared to strictly economic information.

Studies from various geographical areas have found that access to revascularization procedures is reduced in groups with lower SES (22). The population analyzed in this study represents a sample of homogeneously treated patients with STEMI: mostly (>93%) treated by PPCI in a public integrated medical system and features the inclusion of patients not treated with PPCI. The use of PPCI methods in patients with STEMI varies largely by region and can be explained by several factors such as supply factors, the number of physicians, and the number of available hospitalization beds for acute conditions, as well as constraining measures and “patient-level” factors (10). However, the standardization of treatments driven by new evidence-based medicine and consequent guidelines allows countries with different income levels to progressively organize emergency networks for the early management of high-impact diseases in terms of incidence, hospitalization, and case fatality such as in STEMI (23, 24).

Network organization and public access to care and health may result in the neutralization of both higher incidence of the disease and adverse clinical baseline characteristics in low SES groups (25, 26). In our study, the low SES group had a worse cardiovascular profile, longer treatment delays, and different access routes to the health system, which did not, however, translate into worsened 30-day complications or 1-year mortality. Besides, when adjusting for potential confounders, SES data were not associated with either end points, suggesting that a robust healthcare system may flatten socioeconomic baseline disparity potential effect. Similar findings have been described in international cohorts in different studies performed in a single-center fashion (22), in groups of hospitals within a geographical area (20, 26), or within a city (17). Those studies have in common the inclusion of patients with STEMI treated with PPCI as a standard treatment within a structural public STEMI network and showed no interaction of SES with mortality. However, most of these registries reported results from cohorts of patients before 2010. Other previous registries also included patients with other conditions such as non-ST-elevation myocardial infarction (21) or excluded patients treated with fibrinolysis (27). Exclusion of patients treated with fibrinolysis or patients who did not receive PPCI may, however, bias the results of any study aiming to clarify the importance of socioeconomic factors in STEMI prognosis because of the geographical gradient toward the periphery for lower-income groups and its relationship with the optimal timing of treatment for PPCI (17, 28, 29).

The lower tendency of use of DESs in the low-SES group observed in our study has also been reported in American cohorts (30–32), which is a “treatment-risk paradox” in which higher-risk patients are not treated with optimal or evidence-based strategies, and the use of mechanical support devices is reduced, and it may result in prognosis worsening (19, 33, 34). This finding may reflect the fact that differences in outcomes may not only be related to the use of a specific treatment/device in the acute period but to a whole standardized medical approach in which access to secondary prevention may be of utmost importance (35) and that the health system may override the burden of higher risk subsets.

Our study has several limitations. First, the data are derived from the first 7 years of implementation of a regional STEMI network, and its registry has evolved over the years and includes progressively more detailed data such as treated hypertension, treated dyslipidemia, smoking status, previous stroke, number and type of stents, and LV function. Moreover, there were no available records on the medical treatment administered during the index admission, at discharge, and/or regarding other therapies during follow-up such as cardiovascular rehabilitation. These treatments as well as the patients' adherence to prescriptions have been shown to have a direct impact on mortality (35, 36) and may also be related to socioeconomic conditions (21). Finally, the FIRB is a composite index that aggregates data from all inhabitants of a given district, so it does not allow analyzing effects of individual-level SES. However, the FIRB as a surrogate of SES by zip code results in a useful tool. Several prior studies have validated this approach of imputing individual SES in epidemiologic studies reflecting aggregate characteristics of a population and prevailing habits as well as its environmental attributes (such as available health resources) that impact its residents' health (37, 38).

## Conclusion

Barcelona citizens with lower SES who are admitted with STEMI show more unfavorable clinical and cardiovascular risk factor features than citizens with higher SES. Nevertheless, the “Codi IAM” emergency network for STEMI management documented similar 30-day complications and 1-year mortality regardless of SES suggesting that a robust healthy system may override baseline adverse patients' characteristics.

## Data Availability Statement

The datasets presented in this article are not readily available because the dataset belongs to Departament de Salut, Generalitat de Catalunya, and the researchers only have access to statistical analysis.

## Ethics Statement

The studies involving human participants were reviewed and approved by Hospital del Mar Ethics Committee. The patients/participants provided their written informed consent to participate in this study.

## Author Contributions

HT-M: study management, data collection, interpretation of collected data, and preparation of the manuscript. BV: critical revisions of the manuscript. JM: participation in study management and acquisition of data and revision of the manuscript. RL, AA, and NF: critical revisions of the manuscript and data collection. XC: revision of the manuscript and data collection. JG-P, AR, MC, AC-V, XD, NR, and HC-G: revision of the manuscript. PP: extensive revision of the manuscript. JM: study management, interpretation of collected data, statistical analysis, and extensive revision of the manuscript.

## Funding

This project was supported by the CIBERCV of research on cardiovascular diseases personnel hired with its resources.

## Conflict of Interest

The authors declare that the research was conducted in the absence of any commercial or financial relationships that could be construed as a potential conflict of interest.

## Publisher's Note

All claims expressed in this article are solely those of the authors and do not necessarily represent those of their affiliated organizations, or those of the publisher, the editors and the reviewers. Any product that may be evaluated in this article, or claim that may be made by its manufacturer, is not guaranteed or endorsed by the publisher.

## Abbreviations

ACS: Acute coronary syndrome
BMS: bare-metal stent
CHD: coronary heart disease
DES: drug-eluting stent
ECG: electrocardiogram
EMS: emergency medical system
FIRB: Family Income Ratio of Barcelona
OSs: onset of symptoms
PCI: percutaneous coronary intervention
PPCI: primary percutaneous coronary intervention
SES: socioeconomic status
STEMI: ST-elevation myocardial infarction
TIMI: thrombolysis in myocardial infarction.
