# Supplementary material for: Socioeconomic Status and Prognosis of Patients With ST-Elevation Myocardial Infarction Managed by the Emergency-Intervention “Codi IAM” Network
Source: Front Cardiovasc Med. 2022 Apr 25;9:847982. doi: 10.3389/fcvm.2022.847982 (PMC9082814; doi:10.3389/fcvm.2022.847982)
Supplement: Supplementary file 1 [file Data_Sheet_1.docx]

**Supplementary material 1.** Treated STEMI patients treated in Barcelona city by year and socioeconomic status (SES).

|  | **All Patients** | | | **Low SES** | **Mid SES** | **High SES** |
| --- | --- | --- | --- | --- | --- | --- |
| ***Year*** | *Total Barcelona inhabitants* | *Number of STEMI hospitalized patients* | *Hospitalized patients rate per 100.000 inhabitants* | *Hospitalized patients rate per 100.000 inhabitants and 95%CI* | *Hospitalized patients rate per 100.000 inhabitants and 95%CI* | *Hospitalized patients rate per 100.000 inhabitants and 95%CI* |
| **2010** | 1,619,337 | 456 | 28 | 31.6(27.5;36.4) | 26.2(22.8;30.1) | 25.4(19.3;32.2) |
| **2011** | 1,615,448 | 450 | 27 | 27.7(24.0;31.9) | 26.0(22.4;30.2) | 32.9(26.5;40.7) |
| **2012** | 1,620,943 | 459 | 28 | 29.6(25.7;34.2) | 26.3(22.8;30.3) | 30.2(24.4;37.6) |
| **2013** | 1,611,822 | 488 | 30 | 29.5(25.7;33.9) | 32.4(28.6;36.9) | 24.9(19.2;32.4) |
| **2014** | 1,602,386 | 478 | 29 | 38.3(33.6;43.7) | 22.0(18.8;25.6) | 33.3(26.8;40.7) |
| **2015** | 1,604,555 | 509 | 31 | 36.2(31.8;41.3) | 30.6(26.8;34.6) | 24.8(19.4;31.5) |
| **2016** | 1,608,746 | 482 | 29 | 38.3(33.5;43.7) | 24.7(21.5;28.5) | 26.9(21.4;33.9) |
| **2010-2016** | 11,283,237 | 3,322 | 29 | 33.0(28.5;37.5) | 26.7(23.0;30.6) | 28.1(22.2;35.2) |

**Supplementary material 2**. Clinical, procedural and endpoints by DES/BMS use.

|  | DES use  N= 460 | Non-DES use  N= 210 | *P*-value |
| --- | --- | --- | --- |
| **Age,years** | 63.3 (12.5) | 70.7 (14.4) | <0.005 |
| **Female gender, %** | 25.7 | 29.0 | 0.360 |
| **SES,**  **Median+-IQR**  **Percentage into lower SES (<80), %**  **Percentage into middle SES (81-125), %**  **Percentage into higher SES (>126), %** | 81.6  (68.3 – 98.6)  42.6  44.6  12.8 | 80.6  (61.0 – 101.9)  49.5  39.0  11.4 | 0.027  0.250 |
| **Smoker*, %** | 36.5 | 22.9 | <0.005 |
| **High Blood Pressure*, %** | 44.3 | 50.0 | 0.170 |
| **Dyslipidemia*, %** | 36.3 | 35.7 | 0.880 |
| **Diabetes Mellitus, %** | 23.5 | 21.9 | 0.650 |
| **Previous stroke*, %** | 1.5 | 3.3 | 0.130 |
| **Chronic oral anticoagulant*, %** | 0.9 | 3.3 | 0.020 |
| **Previous antiplatelet treatment*, %** | 6.5 | 9.5 | 0.170 |
| **Killip III-IV at presentation, %** | 11.8 | 14.8 | 0.280 |
| **First medical contact**  **General Practitioner, %**  **Emergency Medical System (EMS), %**  **Non- PPCI centre, %**  **PPCI centre, %** | 19.0  49.0  9.8  22.1 | 14.3  50.0  10.0  25.7 | 0.440 |
| **LBBB, %** | 1.3 | 0.5 | 0.330 |
| **Oral intubation and mechanical ventilation, %** | 6.7 | 6.7 | 0.970 |
| **Ventricular Fibrillation, %** | 8.3 | 5.7 | 0.240 |
| **Atrial Fibrillation, %** | 1.3 | 1.9 | 0.550 |
| **AV Blockade, %** | 4.6 | 8.6 | 0.040 |
| **Pulmonary oedema**, %** | 2.0 | 3.3 | 0.280 |
| **Shock, %** | 7.0 | 5.7 | 0.550 |
| **Initial TIMI 0**, %** | 35.4 | 26.7 | 0.025 |
| **Initial TIMI 3**, %** | 9.3 | 8.6 | 0.750 |
| **Final TIMI 0**, %** | 100.0 | 9.5 | 0.310 |
| **Final TIMI 3**, %** | 97.4 | 96.2 | 0.400 |
| **3-vessel disease**, %** | 15.4 | 15.2 | 0.950 |
| **Left main disease**, %** | 4.8 | 2.9 | 0.200 |
| **Anterior STEMI, %** | 49.3 | 29.0 | <0.005 |
| **Inferior STEMI , %** | 41.7 | 60.0 | <0.005 |
| **Bleeding requiring transfusion, %** | 0.0 | 0.5 | 0.310 |
| **OS-First assistance, min** | 60 (35–148) | 90 (40–225) | 0.016 |
| **OS-ECG, min** | 72 (43 – 163) | 105 (52.5–263.5) | 0.002 |
| **ECG-open artery, min** | 80 (63 – 110) | 90 (71 – 115) | 0.002 |
| **OS-Arrival to hospital, min** | 122 (80–220) | 150 (95–317.2) | 0.002 |
| **OS-open artery,min** | 169 (121– 278) | 203 (144.5– 383.5) | < 0.005 |
| **PPCI in less than 120 min, from ECG %** | 80.0 | 76.9 | 0.370 |
| **30-day mortality, %** | 5.0 | 11.0 | 0.005 |
| **30-day composite, %** | 16.7 | 23.3 | 0.043 |
| **One-year mortality in 30-day survivors, %** | 2.7 | 7.5 | 0.007 |

LBBB: Left Bundle Branch Blockade; ECG: electrocardiogram; PPCI: Primary Percutaneous Coronary Intervention.; AV Blockade: Atrio Ventricular Blockade. PPCI: Primary Percutaneous Coronary Intervention. TIMI: Thrombolysis In Myocardial Infarction Flow; BMS: Bare Metal Stent; .DES: Drug Eluting Stent.  STEMI: ST Elevation myocardial infarction; OS: Onset of symptoms. ECG: Electrocardiogram; min: minutes: median [Interquartilic range]

(*) Data available since 2015

(**) Data available since 2012
